# Increased body fat is associated with potentiation of blood pressure response to hypoxia in healthy men: relations with insulin and leptin

**DOI:** 10.1007/s10286-015-0338-2

**Published:** 2016-01-18

**Authors:** Bartłomiej Paleczny, Agnieszka Siennicka, Maciej Zacharski, Ewa Anita Jankowska, Beata Ponikowska, Piotr Ponikowski

**Affiliations:** Department of Physiology, Wroclaw Medical University, ul. Chałubińskiego 10, 50-368 Wroclaw, Poland; Department of Cardiology, Centre for Heart Diseases, 4th Military Hospital, Wroclaw, Poland; Department of Biochemistry, Pharmacology and Toxicology, Wroclaw University of Environmental and Life Sciences, Wroclaw, Poland; Laboratory for Applied Research on Cardiovascular System, Department of Heart Diseases, Wroclaw Medical University, Wroclaw, Poland; Department of Heart Diseases, Wroclaw Medical University, Wroclaw, Poland

**Keywords:** Peripheral chemoreflex, Leptin, Insulin, Autonomic nervous system, Adipose tissue

## Abstract

**Background:**

Increased peripheral chemosensitivity (PChS) has been proposed as mechanism underlying obesity-related sympathoactivation, with insulin and/or leptin as possible mediators. However, human data on PChS in obesity are scarce. Therefore, we explored this issue in a sample of 41 healthy men aged 30–59 years, divided according to body fat percentage (fat %) into two groups: <25 and ≥25 %.

**Methods:**

PChS was assessed using transient hypoxia method [respiratory (PChS-MV), heart rate (PChS-HR), and blood pressure (PChS-SBP) responses were calculated]. Baroreflex sensitivity (BRS-Seq) was assessed using sequence method. Fasting plasma insulin and leptin levels were measured. Homeostatic model assessment (HOMA) was used to assess insulin sensitivity/resistance.

**Results:**

Individuals with ≥25 % body fat demonstrated increased PChS-SBP (*p* < 0.01), but unchanged PChS-MV and PChS-HR (both *p* > 0.4). PChS-SBP was related positively with anthropometric characteristics (e.g. waist circumference, fat %), plasma insulin and HOMA (all *p* < 0.05), and negatively with BRS-Seq (*p* = 0.001), but not with plasma leptin (*p* = 0.27).

**Conclusions:**

In healthy men, overweight/obesity is accompanied by augmented blood pressure response from peripheral chemoreceptors, while respiratory and heart rate responses remain unaltered. Hyperinsulinaemia and insulin resistance (but not hyperleptinaemia) are associated with augmented pressure response from chemoreceptors.

**Electronic supplementary material:**

The online version of this article (doi:10.1007/s10286-015-0338-2) contains supplementary material, which is available to authorized users.

## Background

Hyperactivity of peripheral chemoreceptors has been recently proposed as a pathophysiological mechanism underlying sympathetically mediated diseases [1–3]. Obesity is associated with chronically elevated sympathetic activity which unfavorably affects the cardiovascular system [4]. Thus, it would be tempting to hypothesize deranged reflex responses from peripheral chemoreceptors in obese subjects [5]. In fact, human data on the activity of peripheral chemoreceptors in obesity are scarce, and usually related with respiratory, but not hemodynamic responses from chemoreceptors [6–10].

In humans, peripheral chemoreceptors are mainly located in the carotid bodies [11]. Considering carotid body-mediated mechanisms of obesity-related sympathoactivation, insulin and leptin seem to be of particular relevance since obesity is usually accompanied by hyperinsulinemia and hyperleptinemia [12], and both, insulin and leptin have been shown to exert sympathoexcitatory effects via direct action on the carotid bodies in animal models [13–16]. Surprisingly, to the authors’ knowledge there are no human data on relations between peripheral chemoreflex sensitivity (chemosensitivity) and insulin and leptin plasma levels.

Therefore, the study was aimed to explore the peripheral chemoreflex sensitivity in healthy males across a broad range of adipose tissue mass. More specifically, we attempted: (1) to compare chemosensitivity between two groups of subjects, with normal and increased body fat content, and (2) to explore possible relations between chemosensitivity and plasma levels of insulin and leptin in the entire population studied. To provide more insight into the effects of hyperinsulinemia and hyperleptinemia against a background of autonomic nervous system activity in obesity, heart rate variability and cardiac baroreflex sensitivity were also evaluated.

### Study population

Forty-one healthy males aged 30–59 years volunteered for the study. The rather narrow age range was selected as we [17] and others [18, 19] have reported that the peripheral chemoreflex function in humans deteriorates with age. All subjects were nonsmokers, normotensive, and none was taking any medications or reported any history of acute or chronic illness or alcohol or drug addiction. The study was conducted at the Department of Cardiology, 4th Military Hospital in Wroclaw (Poland). The study protocol was approved by the local Institutional Ethics Committee (Komisja Bioetyczna, Wroclaw, Medical University). All subjects gave informed consent in writing. The study was conducted in accordance with the latest review of the Declaration of Helsinki.

### Study protocol

All subjects were asked to refrain from caffeine and alcohol for at least 12 h before the study. Venous blood samples were collected in the morning (between 7:00 and 9:00) after overnight fasting. Anthropometric characteristics: height (cm), weight (kg), waist circumference (cm) and hip circumference (cm) were measured using the standard methods. Body mass index (BMI, kg/m^2^) and waist-to-hip ratio (WHR) were calculated according to the standard formulae (BMI = weight in kilograms/height in meters squared, WHR = waist circumference in centimeters/hip circumference in centimetres). Body fat percentage (%) was estimated using a multi-frequency bioelectrical impedance analyser (Quadscan 4000, Bodystat Ltd., Douglas, UK). Measurement was carried out with subject lying supine on a flat, nonconductive bed, for at least 15 min.

Study population was divided according to body fat percentage into two groups: with normal and with increased body fat content (i.e. with body fat percentage <25 and ≥25 %, respectively). Unlike for BMI, there are no commonly accepted cut-offs of body fat percentage for classifying individuals as being overweight or obese. The definition of obesity proposed by *The American Society of Endocrinologist* is the body fat percentage of >25 % in men [20]. Similar values have been reported in some large population-based studies (e.g. 25.9 %—the optimum threshold for identifying men at increased cardiometabolic risk, defined as the presence of metabolic syndrome and/or diabetes in the study by Lamb et al. [21]) we decided to set the cut-off of body fat percentage at the level of 25 %.

After blood sampling and anthropometric measurement, autonomic testing was performed in a quiet, light-attenuated room with stable ambient temperature (~22 °C). Autonomic testing included: resting stage and transient hypoxia stage, and was carried out with subject lying supine and attached to the equipment described below (see below, section: Study equipment).

### Laboratory measurements

Fasting plasma glucose concentration (mmol/L) was measured using colorimetric assay with glucose oxidase (Konelab Prime 60i/30i; Thermo Fisher Scientific, Waltham, MA). Fasting plasma insulin level (μU/mL) was measured using chemiliminescence on LIAISON Analyzer (DiaSorin S.p.A., Saluggia, Italy). Fasting plasma leptin level (ng/mL) was measured using ELISA (LDN Labor Diagnostika Nord GmbH and Co. KG, Nordhorn, Germany). The natural logarithms (ln) of serum insulin and leptin were calculated and used in the analyses.

Insulin sensitivity/resistance was measured using the homeostasis model assessment (HOMA-IR), according to the formula: HOMA-IR = fasting plasma insulin (μU/mL) × fasting plasma glucose (mmol/L)/405 [22].

### Study equipment

Non-invasive, continuous recording of hemodynamic parameters: systolic blood pressure (SBP, mmHg), diastolic blood pressure (DBP, mmHg), mean arterial pressure (MAP, mmHg), stroke volume (SV, mL/beat), cardiac output (CO, L/min) and systemic vascular resistance (SVR, dyn × s/cm^5^) were obtained using Nexfin device (BMEYE B.V., Amsterdam, Netherlands). ECG recording was performed using ECG module for Nexfin device. Heart rate (HR, bpm) was calculated from ECG recording.

Minute ventilation (MV, L/min) was calculated based on instantaneous values of breathing rate and tidal volume, measured continuously with a differential pressure transducer (FE141 Spirometer; ADInstruments, Sydney, NSW, Australia) and a breathing circuit consisting of an oronasal face mask (Hans Rudolph, Inc., Shawnee, KS, USA) with two-way non-rebreathing *T*-shape valve (Hans Rudolph) and a 1000 L/min flowhead (MLT3000L; ADInstruments) on the expiratory side of the valve. Continuous measurement of end-tidal carbon dioxide concentration (etCO_2_, mmHg) was provided by a capnograph (Capstar 100, CWE Inc., Ardmore, PA, USA). Blood oxygen saturation (SpO_2_, %) was measured continuously using an ear probe connected to a pulse oximeter (Masimo Corporation Irvine, CA, USA).

During the transient hypoxia stage, pure nitrogen was administrated from 10-L gas cylinder via small-diameter tube placed inside large-diameter breathing tube (ADInstruments) connected directly with a face mask. As shown by in the previous studies from our laboratory [17, 23–25], within this breathing circuit, administration of nitrogen with a flow rate >15 L/s (regulated to the nearest 0.1 L) is sufficient to cause a transient nitrogen accumulation in the circuit, and in turn to evoke a substantial, short-lasting desaturation.

All data were recorded and stored at sampling frequency of 1 kHz (16-bit resolution) using data acquisition system (PowerLab 16/30, ADInstruments) and computer (Dell Inc., Round Rock, TX, USA).

The first 3- to 5-min recording preceding the 15-min resting stage was ignored and served only to familiarize the subject with the experimental equipment. Resting values of HR, SBP, DBP, MAP, SV, CO, SVR, MV, etCO_2_, and SpO_2_ were calculated as arithmetic averages from 10-min recording of an acceptable quality isolated from 15-min resting stage recording. The same 10-min recording was used to calculate heart rate variability cardiac baroreflex sensitivity.

### Assessment of heart rate variability

Heart rate variability (HRV) parameters were used to assess the sympathovagal interaction modulating cardiovascular function [26, 27].

The following HRV parameters were calculated: (1) time domain parameters: average of all RR intervals (mean RR, ms); standard deviation of all RR intervals (SDNN, ms); number of pairs of adjacent NN intervals differing by more than 50 ms (NN50 count); (2) frequency-domain parameters: power of HRV spectrum within low frequency range: 0.04–0.15 Hz (LF, ms^2^) and high frequency range: 0.15–0.4 Hz (HF, ms^2^) [26].

Frequency-domain parameters of HRV were calculated using standard autoregressive methods [27]. SDNN reflects overall autonomic modulation of the heart function, whereas NN50 count and HF are considered as markers of primarily vagal tone. The physiological mechanisms underlying LF are less clear and include primarily sympathetic or mixed, sympathetic and parasympathetic influences [26].

### Assessment of cardiac baroreflex sensitivity

Cardiac baroreflex sensitivity (BRS) was evaluated using sequence method [28]. The sequence method is based on the computer identification of spontaneously occurring sequences of three or more consecutive heart beats, characterized by either progressive rise in SBP (by at least 1 mmHg) with concomitant lengthening in RR interval (by at least 5 ms) or progressive fall in SBP (by at least 1 mmHg) with concomitant shortening in RR interval (by at least 5 ms). For each sequence selected, a slope of the regression line relating RR interval to SBP is calculated, and BRS is defined as the average of all slopes for all sequences selected (BRS-Seq, ms/mmHg) [28, 29].

### Assessment of peripheral chemoreflex sensitivity

The transient hypoxia method was used to evaluate a ventilatory [30] and hemodynamic [23, 24] peripheral chemosensitivity. During the transient hypoxia stage, subject was switched briefly from breathing with room-air to breathing with pure nitrogen for 10–45 s. For each subject, this procedure was repeated 5–7 times to achieve falls in SpO_2_ with maximal desaturations varying from 65 to 85 %. The length of subsequent periods of breathing with nitrogen was adjusted ad hoc based on the fall in SpO_2_ observed during the first, 10-s period. Subsequent periods of nitrogen-breathing were separated by at least 5-min periods of room-air breathing to allow for the measured parameters to return to the baseline levels. Two components of hypoxia-induced chemoreflex response were assessed: (1) ventilatory response (increase in minute ventilation) and (2) hemodynamic responses: HR response (increase in HR) and BP response (increase in SBP) using the following calculations.

*Ventilatory response* For each nitrogen-breathing period, three largest consecutive values of MV within 35-s after the end of nitrogen-breathing period were averaged and plotted against an associated nadir of SpO_2_ (providing Point *A*). Similarly, baseline value of MV was plotted against baseline value of SpO_2_ (providing Point *B*). Baseline values of MV and SpO_2_ were defined as averages from 90-s period preceding the nitrogen-breathing period. Peripheral chemosensitivity in terms of ventilatory response (PChS-MV, L/min/SpO_2_ %) was defined as a slope of the regression line relating all Points *A* to all Points *B.*

*Hemodynamic responses* Peripheral chemosensitivity in terms of SBP response (PChS-SBP, mmHg/SpO_2_ %) and HR response (PChS-HR, bpm/SpO_2_ %) were assessed analogously as peripheral chemosensitivity in terms of ventilatory response described above, with two differences: (1) 55-s period instead of 35-s period after nitrogen-breathing period were used, and (2) the highest values of SBP (for PChS-SBP) or HR (for PChS-HR) were used instead of the averaged three largest consecutive values of MV.

Detailed description and validation of this method was presented before [23, 24].

### Statistical analysis

STATISTICA 10 (Statsoft, Tulsa, OK, USA) and MATLAB (MathWorks, Natick, MA, USA) were used to data and statistical analysis. The method of Kolmogorov–Smirnov was used to determine whether the measured parameters followed a normal distribution. Variables were presented as mean ± standard deviation (for normally distributed variables) or median with upper and lower quartile (for non-normally distributed variables). Non-normally distributed variables were logarithmically transformed before statistical analysis (natural logarithm) and proved to be normalized afterwards, before statistical analysis. For all non-normally distributed variables, only log-transformed values (ln) were used in the analyses. Student’s *t* test was used for inter-group comparisons. For the selected, statistically significant inter-group differences, the effect size was calculated using the method proposed by Cohen [31], as standardized difference between two means. Relations between variables were tested in univariate models (*r* Pearson’s linear correlation coefficients) and in multivariate models. *p* < 0.05 was considered statistically significant.

## Results

### Clinical characteristics in subjects with normal vs. increased body fat content

Subjects with increased body fat content were characterized by higher body weight, waist circumference, body mass index, and waist-to-hip ratio (all *p* < 0.001). Respiratory parameters at rest did not differ between groups (all *p* > 0.4). Heart rate, systolic, diastolic and mean arterial pressure, and cardiac output were higher in subjects with increased body fat content (all *p* < 0.05). Higher values of plasma insulin and leptin, and HOMA-IR were found in those subjects (all *p* < 0.05) (Table 1).

**Table 1:** Clinical characteristics in men with normal and increased body fat content

	Body fat percentage <25 % (*N* = 27)	Body fat percentage ≥25 % (*N* = 14)	*p*
*Anthropometric parameters*			
Age (years)	46 ± 8	44 ± 8	0.38
Weight (kg)	81 ± 11	104 ± 16	<0.001
Waist (cm)	88 ± 9	109 ± 10	<0.001
BMI (kg/m^2^)	25.1 ± 2.7	33.7 ± 3.5	<0.001
Fat (%)	18.4 ± 3.4	30.1 ± 3.7	<0.001
WHR	0.90 ± 0.06	1.00 ± 0.05	<0.001
*Respiratory parameters at rest*			
Breathing rate (breaths/min)	12 ± 4	12 ± 4	0.95
Minute ventilation (L/min)	11 ± 3	11 ± 4	0.68
etCO_2_ (mmHg)	36 ± 5	37 ± 3	0.78
SpO_2_ (%)	97 ± 1	96 ± 2	0.42
*Haemodynamic parameters at rest*			
Heart rate (bpm)	65 ± 10	72 ± 7	0.02
Systolic blood pressure (mmHg)	117 ± 13	127 ± 13	0.02
Diastolic blood pressure (mmHg)	68 ± 7	74 ± 9	0.02
Mean arterial pressure (mmHg)	87 ± 10	95 ± 10	0.02
Stroke volume (mL/beat)	101 ± 10	103 ± 14	0.51
Cardiac output (L/min)	6.5 ± 0.8	7.4 ± 0.9	0.002
Systemic vascular resistance (dyn × s/cm^5^)	1090 ± 151	1047 ± 162	0.40
*Blood parameters*			
Fasting plasma glucose (mmol/L)	101 ± 9	106 ± 17	0.31
Fasting plasma insulin (μU/mL)	7.9 (4.5, 10.7)	13.5 (10.6, 22.8)	<0.001
Fasting plasma leptin (ng/mL)	4.9 (3.6, 7.0)	16.6 (7.3, 23.9)	<0.001
HOMA-IR	2.24 ± 1.71	3.95 ± 2.06	0.04

Data are presented as a mean ± standard deviation or median with lower and upper quartile where appropriate *p* values for inter-group comparisons using Student’s *t* test are presented

*Waist* waist circumference, *BMI* body mass index, *Fat* body fat percentage, *WHR* waist to hip ratio, *etCO*_*2*_ end-tidal CO_2_ concentration, *SpO*_*2*_ blood oxygen saturation, *HOMA*-*IR* homeostatic model assessment

### Peripheral chemoreflex sensitivity in subjects with normal vs. increased body fat content

Subjects with increased body fat content displayed greater BP response to hypoxia, as compared with the remaining subjects (*p* = 0.004). The effect size for this difference was large (Cohen’s *d* = 1.04), according to the criteria by Cohen [31]. However, there were no inter-group differences in respiratory and HR responses to hypoxia (both *p* > 0.4) (Table 2).

**Table 2:** Peripheral chemoreflex sensitivity in men with normal and increased body fat content

	Body fat percentage <25 % (*N* = 27)	Body fat percentage ≥25 % (*N* = 14)	*p*
*Peripheral chemoreflex sensitivity*			
PChS-MV (L/min/SpO_2_ %)	0.24 (0.15, 0.40)	0.26 (0.19, 0.33)	0.48
PChS-SBP (mmHg/SpO_2_ %)	0.53 ± 0.28	0.81 ± 0.27	0.004
PChS-HR (bpm/SpO_2_ %)	0.46 ± 0.19	0.50 ± 0.32	0.67

Data are presented as a mean ± standard deviation or median with lower and upper quartile where appropriate; *p* values for inter-group comparisons using Student’s *t* test are presented

*PChS*-*MV* peripheral chemoreflex sensitivity in terms of respiratory response, *PChS*-*HR* peripheral chemoreflex sensitivity in terms of heart rate response, *PChS*-*SBP* peripheral chemoreflex sensitivity in terms of systolic blood pressure response

### Autonomic characteristics in subjects with normal vs. increased body fat content

Parameters of overall HRV (mean RR, SDNN) were reduced in subjects with increased body fat content (all *p* ≤ 0.05), suggesting depressed autonomic regulation of heart function. In line with this notion, lower values of vagally related parameters (NN50 count, BRS-Seq) were found in those subjects. However, there were no differences in frequency-domain parameters of HRV (LF, HF, both *p* > 0.5) (Table 3).

**Table 3:** Autonomic characteristics in men with normal and increased body fat content

	Body fat percentage <25 % (*N* = 27)	Body fat percentage ≥25 % (*N* = 14)	*p*
*Heart rate variability at rest*			
Mean RR (ms)	950 ± 133	831 ± 95	0.007
SDNN (ms)	39 ± 9	33 ± 8	0.04
NN50 count	28 (7, 57)	17 (1, 27)	0.03
LF (ms^2^)	700 (259, 1260)	775 (354, 1203)	0.82
HF (ms^2^)	288 (185, 713)	156 (83, 520)	0.51
*Baroreflex sensitivity at rest*			
BRS-Seq (ms/mmHg)	12.9 ± 6.1	7.7 ± 2.7	0.006

Data are presented as a mean ± standard deviation or median with lower and upper quartile where appropriate; *p*values for inter-group comparisons using Student’s *t* test are presented

*Mean RR* mean duration of RR interval, *NN50 count* number of pairs of adjacent RR intervals differing by more than 50 ms, *LF* low frequency range of heart rate variability spectrum, *HF* high frequency range of heart rate variability spectrum, *BRS*-*Seq* cardiac baroreflex sensitivity assessed by the sequence method

### Correlations between peripheral chemoreflex sensitivity and selected variables

**Fig. 1:**
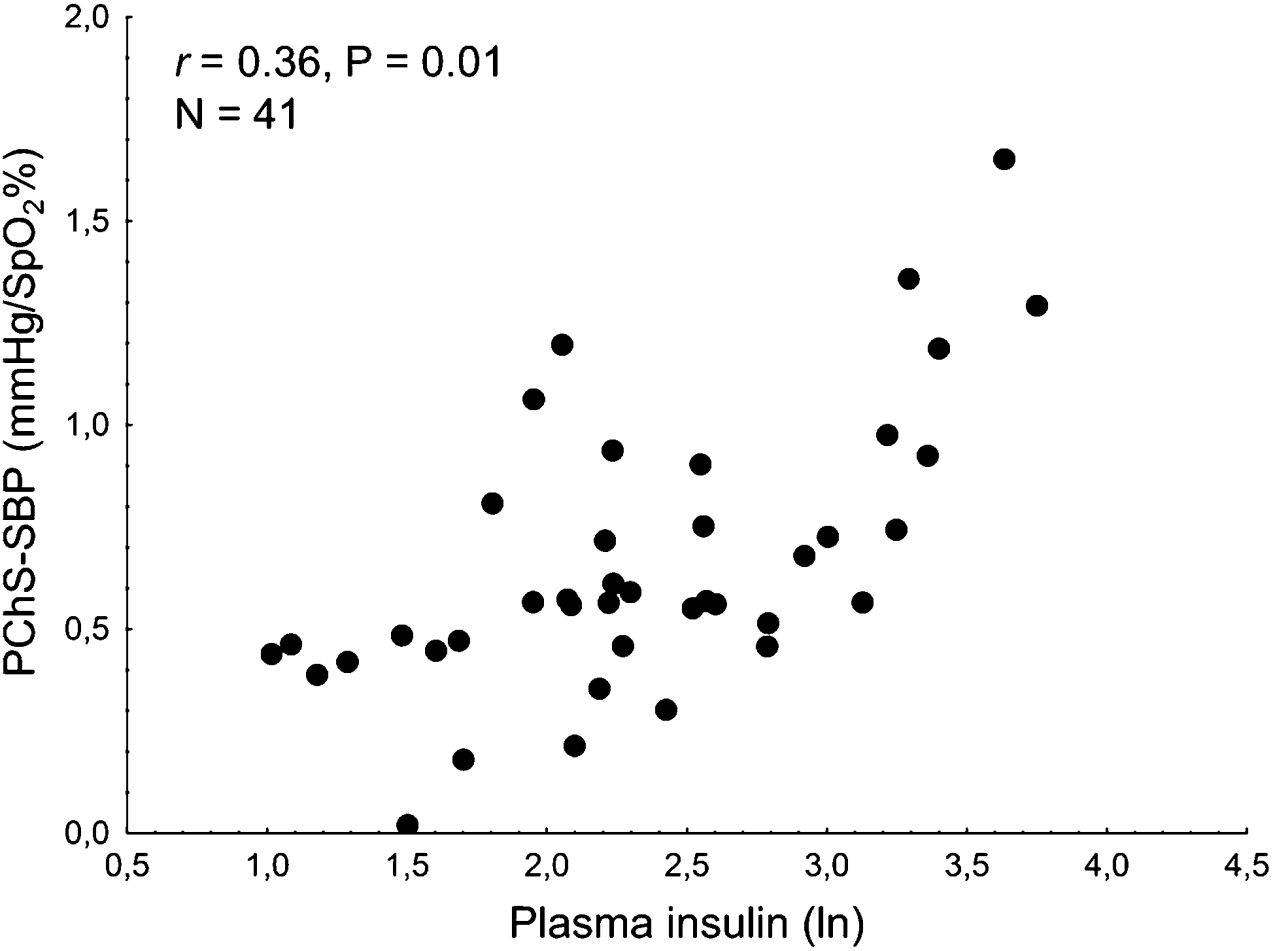
Relation between plasma insulin level (natural logarithm) and blood pressure response to hypoxia in the population studied

To investigate the possible determinants of augmented BP response to hypoxia in subjects with increased body fat content, correlatory analyses have been performed. In the population studied, magnitude of BP response to hypoxia was related positively with some anthropometric characteristics (waist circumference, body fat percentage, waist to hip ratio, all *p* < 0.05), plasma insulin level and HOMA-IR (both *p* < 0.05), and negatively with some HRV parameters (mean RR, SDNN, LF, all *p* < 0.05), and cardiac baroreflex sensitivity (*p* = 0.001). Of importance, plasma leptin level was not related with PChS-SBP (*p* = 0.27) (Table 4, Fig. 1).

**Table 4:** Correlations between blood pressure response to hypoxia and selected variables in the entire population studied

	Age (years)	Weight (kg)	Waist (cm)	BMI (kg/m^2^)	Fat (%)	WHR	Plasma leptin (ln)	Plasma insulin (ln)	HOMA-IR	Mean RR (ms)	SDNN (ms)	NN50 count	LF (ln)	HF (ln)	BRS-Seq (ms/mmHg)
PChS-SBP (mmHg/SpO_2_ %)	0.03	0.25	0.31	0.24	0.34	0.32	0.27	0.36	0.34	−0.43	−0.40	−0.26	−0.40	−0.25	−0.46
	*p* = 0.86	*p* = 0.10	*p* = 0.04	*p* = 0.12	*p* = 0.03	*p* = 0.04	*p* = 0.27	*p* = 0.01	*p* = 0.05	*p* = 0.004	*p* = 0.008	*p* = 0.08	*p* = 0.007	*p* = 0.10	*p* = 0.001

*r* Pearson’s linear correlation coefficients with *p* values are presented

*PChS*-*SBP* peripheral chemoreflex sensitivity in terms of systolic blood pressure response, *Waist* waist circumference, *BMI* body mass index, *Fat* body fat percentage, *WHR* waist to hip ratio, *HOMA*-*IR* homeostatic model assessment, *Mean RR* mean duration of RR interval, *NN50 count* number of pairs of adjacent RR intervals differing by more than 50 ms, *LF* low frequency range of heart rate variability spectrum, *HF* high frequency range of heart rate variability spectrum, *BRS*-*Seq* cardiac baroreflex sensitivity assessed by the sequence method

Multivariate regression model included all the variables that appeared to significantly correlate with PChS-SBP in the univariate models, with the exception of those which were highly interrelated (arbitrary cut-off: *r* = 0.70). Two models have been constructed. In the first model, including waist circumference, BMI, and plasma insulin (ln), only plasma insulin (ln) remained statistically significant (*β* = 0.39, *p* = 0.04; *R*^2^ for the model = 0.25, *p* = 0.02). In the second model, including waist circumference, BMI, and HOMA-IR, only HOMA-IR remained statistically significant (*β* = 0.39, *p* = 0.04; *R*^2^ for the model = 0.27, *p* = 0.03).

## Discussion

The major findings of the study are that: (1) healthy men with increased body fat content display augmented BP response, but unchanged respiratory and HR responses from peripheral chemoreceptors, and (2) elevated plasma insulin level and reduced insulin sensitivity (but not plasma leptin level) are associated with augmented BP response from peripheral chemoreceptors in those subjects.

### Peripheral chemoreflex sensitivity in obesity

Our results suggest that being overweight/obese is accompanied by a selective potentiation of BP response to stimulation of peripheral chemoreceptors with hypoxia.

Literature data on the activity of hemodynamic component of peripheral chemoreflex are scarce. Narkiewicz et al. [10] have not found any significant difference between normal-weight and obese subjects in BP and HR responses to hypoxia. Such results stay in line with our findings in regards to HR response, but not for BP response. This discrepancy may be due to distinct methodological approach. Narkiewicz et al. have used a steady-state hypoxia method, with relatively weak hypoxic stimulus (10 % O_2_ in N_2_) applied for three minutes. Transient (acute) hypoxia method, which was used in our study, includes several short (<1 min) episodes of breathing with pure N_2_, and possibly results is much greater activation of chemoreceptors [32].

Previous studies exploring the activity of respiratory component of peripheral chemoreflex in obesity have provided conflicting results, reporting either increased [7, 33], normal [6], or even decreased [34] ventilatory responsiveness to hypoxia in obese subjects. However, these (rather old) papers suffer from methodological shortcomings (combining data from both sexes, effect of aging or various co-morbidities, e.g. hypertension, obstructive apnea/hypopnea syndrome) [8]. Two well-controlled studies by Narkiewicz et al. [10] and Buyse et al. [8] have found no significant effect of obesity on hypoxic ventilatory response. Such results stay in line with our study, but, as mentioned above, direct comparison of the results may be misleading due to different methodology applied to assess chemosensitivity.

Important strengths of our study need to be emphasized. First, we defined normal and increased body fat based on bioelectrical impedance analysis, instead of body mass index. Second, we assessed both, ventilatory and hemodynamic components of peripheral chemoreflex. Previous studies from our laboratory [17, 24] and experimental data from animal models [35–37] suggest that respiratory, BP and HR responses from peripheral chemoreceptors are conveyed by at least partially distinct pathways, and some pathophysiological processes may disturb one component of the peripheral chemoreflex, leaving the other intact.

The magnitude of potentiation of the pressure response found in our study in obese healthy subjects (mean: 0.81 ± 0.27 mmHg/SpO_2_ %) is in the range of reported for patients with chronic systolic heart failure (CHF) (Niewinski et al. [23]: mean: 0.67 mmHg/SpO_2_ %) or arterial hypertension (Tubek et al., unpublished: mean: 1.06 mmHg/SpO_2_). A growing body of evidence suggests that the hypersensitivity of the carotid bodies contributes to increased sympathetic tone in certain cardiovascular diseases, and surgical removal of the carotid body may be considered as a novel therapeutic approach aimed to restore autonomic balance in those patients [3]. Our finding of the enhanced pressure response to hypoxia in obese subjects indicates that similar mechanism may be involved in the development of obesity-related sympathoactivation, as proposed by Porzionato [5]. Interestingly, however, we did not observe a parallel increase in ventilatory chemosensitivity in obese subjects. The reason of such discrepancy remains elusive and further studies are needed.

### Relations between insulin/leptin and peripheral chemoreflex sensitivity in obesity

Both, insulin and leptin have been proposed as key mediators triggering sympathetic overactivity in obese subjects via direct action on the peripheral chemoreceptors in the carotid bodies [5, 15]. However, possible relations between both, plasma insulin and leptin levels and peripheral chemoreceptors sensitivity have not been investigated in humans. We have shown, for the first time, in a large sample of healthy males across a broad range of adipose tissue mass that plasma insulin level, but not leptin level is related positively with BP responsiveness of peripheral chemoreceptors.

The sympathoexcitatory effects of insulin are well established [38]. Acute hyperinsulinemia has been shown to increase plasma catecholamine concentration [39], plasma norepinephrine spillover [40], and muscle sympathetic nerve activity (MSNA), measured directly using microneurography technique [41, 42] in humans. Recent study by Ribeiro et al. [15] on rats has raised the possibility that carotid bodies play an important role in mediating the sympathoexctitatory effects of insulin. The authors were first to identify insulin receptors in the rat carotid bodies, and to demonstrate, that insulin administration increases activity of the carotid bodies, initiating a release of neurotransmitter and leading to hyperventilation. Even more importantly, in this study bilateral carotid body resection has prevented the development of hypertension and insulin resistance in rats on hypercaloric diet, thereby emphasizing a physiological significance of this mechanism.

A growing body of evidence supports the sympathoexcitatory effects of leptin [43]. In animals, intravenous [44–46] or central [42–49] leptin infusion caused marked sympathoactivation, and this was accompanied by hemodynamic consequences (increase in BP and HR) in some [45], but not all studies [46]. In humans, hyperleptinaemia has been reported to correlate positively with sympathetic activity, as assessed by heart rate variability [46, 50], plasma norepinephrine spillover [52], and direct microneurographic recordings [53], and genetically based human leptin deficiency is accompanied by substantially attenuated sympathetic tone [54]. Recently, Machleidt et al. [55] have shown that, in lean healthy men, acute intravenous infusion of leptin directly increases MSNA. Direct role for the carotid bodies in mediating sympathoexcitatory effects of hyperleptinaemia has been proposed [5], as leptin receptors have been identified in rat and human glomus cells [16], and intravenous infusion of leptin have been reported to activate these cells [13, 14]. Microinjection of leptin into the nucleus tractus solitarii has potentiated BP response to stimulation of peripheral chemoreceptors in rats [56].

Of relevance, virtually all studies investigating the autonomic effects of leptin have ignored a possible impact of plasma insulin [50, 51, 53, 57].

Also, depressed cardiac baroreflex sensitivity found in men with increased body fat content may be considered as a mechanism underlying potentiated response to hypoxia in these subjects. This possibility cannot be excluded, especially in the light of robust negative relation between PChS-SBP and BRS-Seq found in this study. However, experimental data suggests that, during and after hypoxia, baroreflex is resetted toward higher levels of BP [58, 59], and thereby does not abolish the hypoxia-induced rise in BP.

### Autonomic control of the heart in obesity

Reduced vagal outflow to the heart, and blunted cardiac baroreflex sensitivity are well-established hallmarks of human and experimental animals obesity [60–62], and have been confirmed in the present study. Of note, lack of concomitant reduction in LF—a marker of sympathetic control of the heart—is in agreement with other reports suggesting that sympathetic outflow is controlled in a tissue specific manner, and cardiac sympathetic modulation is unchanged or even reduced in obesity, despite generalized sympathetic activation [4, 63].

### Study limitations

We acknowledge that our study is not without limitations. First, in this study design no cause-effect relationship would be drawn. Second, for uniformity, only male subjects were enrolled in the study. Therefore, we acknowledge that our results cannot necessarily be extrapolated to women. Third, the concentration of the soluble leptin receptor was not measured. Such information would increase insight into the ability of leptin to penetrate blood–brain barrier, and to act on the central nervous system structures [64]. Fourthly, a direct measurement of MSNA using microneurography technique [65] would provide a better estimation of sympathovagal balance than indirect methods as heart rate variability [66]. Given the observed clustering of obesity, hypertension, inflammation, intermittent hypoxia, hyperglycemia, and insulin and leptin resistance, studies in humans in this area are extremely difficult and require innovative experimental approaches.

The fact that the magnitude of the pressure response to hypoxia was correlated with body fat mass, but not with plasma leptin appears counterintuitive, as leptin is produced in direct proportion to the amount of adipose tissue, and robust, linear relation between body fat mass and plasma leptin is usually observed [12]. Such relation was also found in our study [fat % vs. plasma leptin (ln), *r* = 0.67, *p* < 0.001]. From the physiological perspective, at least two explanations may be considered: (1) other fat-related factors (according to the results of the multiple regression—mainly, plasma insulin or insulin sensitivity) seem to contribute more to the pressure response to hypoxia than plasma leptin level, (2) altered sensitivity of leptin receptors in the carotid body may distort a possible relation between pressure response to hypoxia and plasma leptin level.

The study adds another important piece to the complex influence of insulin and leptin on autonomic reflex control of cardiorespiratory function. Both, insulin and leptin have been considered the key players linking obesity, autonomic derangement, and sympathetically mediated diseases (e.g. hypertension). Nevertheless, the exact mechanisms of its action on autonomic nervous system function remain elusive and further detailed studies in this area are required.

## Electronic supplementary material

Below is the link to the electronic supplementary material.

Supplementary material 1 (JPEG 52 kb)
