# Supplementary figures and images for: Increased body fat is associated with potentiation of blood pressure response to hypoxia in healthy men: relations with insulin and leptin
Source: Clin Auton Res. 2016 Jan 18;26:107–16. doi: 10.1007/s10286-015-0338-2 (PMC4819928; doi:10.1007/s10286-015-0338-2)

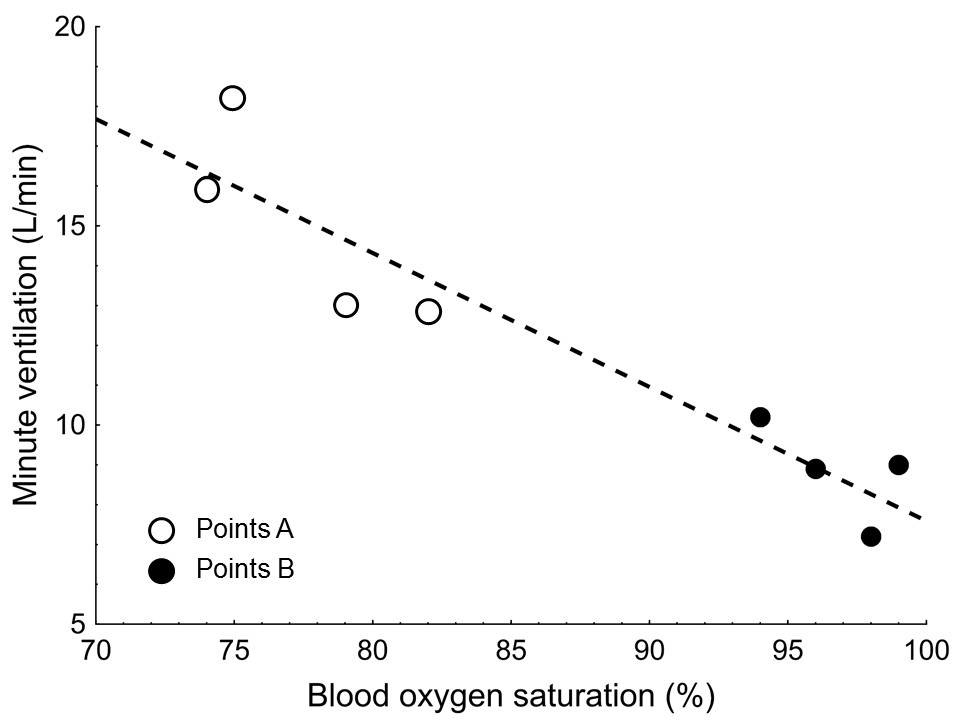

Supplement: Supplementary file 1 — Supplementary material 1 (JPEG 52 kb) [file 10286_2015_338_MOESM1_ESM.jpg]
